# Automated data extraction—A feasible way to construct patient registers of primary care utilization

**DOI:** 10.3109/03009734.2011.653015

**Published:** 2012-02-15

**Authors:** Mats Martinell, Jan Stålhammar, Johan Hallqvist

**Affiliations:** Department of Public Health and Caring Sciences, Uppsala University, Uppsala, Box 564, SE 751 22, Uppsala, Sweden

**Keywords:** Data extraction, data mining, electronic medical records (EMRs), knowledge discovery in databases (KDD), primary health care

## Abstract

**Introduction.:**

Electronic medical records (EMRs) enable analysis of health care data by using data mining techniques to build research databases. Though the reliability of the data extraction process is crucial for the credibility of the final analysis, there are few published validations of this process. In this paper we validate the performance of an automated data mining tool on EMR in a primary care setting.

**Methods.:**

The Pygargus Customized eXtraction Program (CXP) was programmed to find and then extract data from patients meeting criteria for type 2 diabetes mellitus (T2DM) at one primary health care clinic (PHC). The ability of CXP to extract relevant cases was assessed by comparing cases extracted by an EMR integrated search engine. The concordance of extracted data with the original EMR source was manually controlled.

**Results.:**

Prevalence of T2DM was 4.0%, which correspond well to previous estimations. By searching for drug prescriptions, diagnosis codes, and laboratory values, 38%, 53%, and 91% of relevant cases were found, respectively. The sensitivity of CXP regarding extraction of relevant cases was 100%. The specificity was 99.9% due to 12 non-T2DM cases extracted. The congruity at single-item level was 99.6%. The 13 incorrect data items were all located in the same structural module.

**Conclusion.:**

The CXP is a reliable and accurate data mining tool to extract selective data from EMR.

## Introduction

Primary care manages the unselected health panorama as well as a majority of chronic diseases of the population (1). Historically, primary care data have been scarcely accessible due to their geographical dispersion and large volume, but with the introduction of electronic medical records (EMRs) possibilities have emerged to condense and extract information for scientific research. Since 2005 more than 95% of primary health care centres (PHCs) in Sweden use EMRs for their documentation (2). Principally EMR data may be stored in two ways: either as *narrative* data, organized by subheadings (e.g. blood pressure, weight, smoking habit), or as *structured* data, which are basically separate modules within the EMR (e.g. laboratory results, prescriptions, and diagnoses) (3). Most EMR systems have an integrated search engine for *structured* data but not for *narrative* data. Until recently the only way to include *narrative* data into registers was by manual inputting of data (4–8). The Pygargus Customized eXtraction Program (CXP) is an extraction tool that searches *structured* data to find relevant cases and extracts relevant *narrative* data. The CXP has been used to generate registers to study treatment patterns (9–11), implementation of guidelines (12–14), health economic analyses (15,16), and head-to-head comparisons of pharmaceuticals (17) in a real-life primary care setting. In this paper we will evaluate the ability of CXP to extract relevant EMRs and the quality (coherence and occurrence) of extracted data in comparison with the original EMRs.

## Material and methods

### Material

The CXP is designed to extract data from EMR provided by Profdoc^™^. The extraction process is conducted in two steps. In the first step inclusion criteria are programmed from 1) International Classification of Diseases (ICD-9 or ICD-10), 2) prescription (ATC code), or 3) laboratory values. The EMRs that meet the criteria for step 1 are given a personal identification number (PIN). In step 2 the CXP is programmed (customized) to extract specified data, as given in Table I. The final CXP output is a database of PINs (step 1) that contains data (step 2) organized in nine tables (eight *structural* and one *narrative*), Table I. The study was approved by the Regional Ethical Review Board of Uppsala, Sweden (Dnr. 2005:174).

**Table I.** The CXP arranged extracted data into nine tables that correspond to the nine modules in the original EMR. Data in the top eight tables are extracted from *structural* data, whereas “Terminology” is extracted from *narrative* data.

Name of table	Data to be customized for second step in the extraction process
Contacts	Type of contact registration (e.g. doctor, nurse, telephone, administrative), user ID, and date or time interval of contact
Diagnosis	Name, code according to ICD –9 –10, and date or time interval of diagnosis
Biometrics	Weight, height, and BMI sorted by date
Documents	Referrals and other documentation sorted by date
Drugs	Prescriptions (name, ATC code, iteration, and dosage) sorted by date
Biochemical analysis	Analyses, values, and units sorted by date
Measurement	Biometric measurements documented outside the table “Terminology”
Patients	Gender, age, alive/dead
Terminology	*Narrative* data in the core journal text are found under selected subheadings (e.g. current disease, heritage, physical status)

### Study site

The EMR server used for this evaluation was located at the Eriksberg PHC in Uppsala, Sweden. The PHC utilized Profdoc^™^ EMR system between 1993 and 2005. During this period 10,753 EMRs had been opened.

### Ability of CXP to extract prevalent cases

Profdoc^™^ EMRs contain an integrated extraction tool called eXtractor^™^. It has the same functionality as the first step of the CXP extraction process but cannot perform the second step. Since eXtractor^™^ is an integrated component of the EMR, we used it as reference to evaluate the ability of the CXP to identify patients with type 2 diabetes mellitus (T2DM). Both the eXtractor^™^ and the CXP were programmed to identify EMRs that met at least one of the following criteria: 1) International Classification of Diseases (ICD-9 and ICD-10) diagnostic code of T2DM, 2) prescription of an oral antidiabetic agent (ATC code), or 3) fasting blood or plasma glucose concentration indicative of T2DM according to the WHO classification.

### Quality of data extracted by the CXP

**Figure 1.**
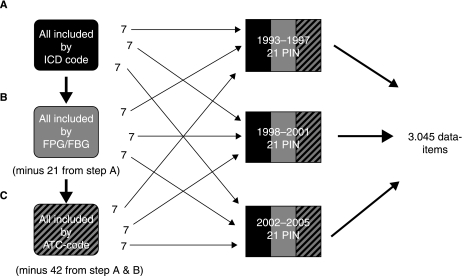
Flow chart for the procedure of selecting personal identification numbers (PIN) to assess the congruity of CXP extracted data to the original EMRs. In step B, the PIN already selected in step A was excluded. In step C, PINs from step A and step B were excluded. Altogether 3,045 data items were compared.

To evaluate step 2 of the CXP extraction process the quality (coherence and occurrence) of data extracted by the CXP was manually compared to the original EMR data. An external consultant was appointed to select which PINs were to be controlled. First, all PINs included by diagnosis (ICD code) were shuffled in a randomized manner. PINs 1–7 were selected for period 1993 to 1997, PINs 8–14 for 1998 to 2001, and PINs 15–21 for 2002 to 2005. The procedure was then repeated for laboratory fasting plasma glucose (FPG) or fasting blood glucose (FBG) concentration and for drug prescription (ATC code) (steps B and C, Figure 1). Then all data items in selected PINs were controlled by occurrence and coherence to the original EMR. All selected PINs contained data from all nine tables described above (Table I).

## Results

### Efficacy of CXP to extract prevalent cases

**Figure 2.**
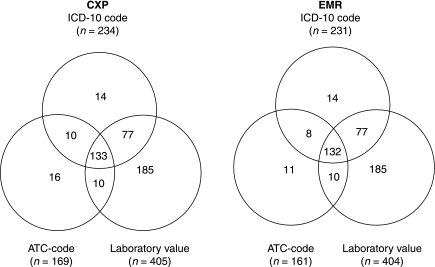
Venn diagrams for data extraction by CXP and by eXtractor, illustrating distribution of inclusion criteria (ICD-10 code, ATC code, laboratory value) for each patient. Patients extracted by Pygargus CXP (*n* = 445) and by eXtractor (*n* = 433).

The CXP identified a subset of 445 EMRs fulfilling at least one of the three inclusion criteria. They were distributed as follows: 234 patients by ICD-10 code, 169 patients by ATC code, and 405 patients by laboratory values. By only using laboratory values as inclusion criteria 91% of the cases were included (Figure 2).

The EMR integrated search engine identified 433 cases, distributed as follows: 231 (53.3%) by ICD-10 code, 161 (37.2%) by ATC code, and 404 (93.3%) by laboratory values. An almost total congruity was found between the EMR integrated search engine and CXP. The sensitivity of the extraction was 100% and specificity 99.9%. The positive prediction value for CXP was 97.3%, and the negative prediction value was 100%.

The CXP found 12 more cases than the EMR integrated search engine when using drug prescription (ATC code). A manual deletion of inaccurate prescriptions by the caregiver was most likely the cause. According to the developer (Pygargus) the CXP does not recognize such manoeuvres in contrast to the integrated search engine (eXtractor).

### Congruity of extraction on a data item level

**Figure 3.**
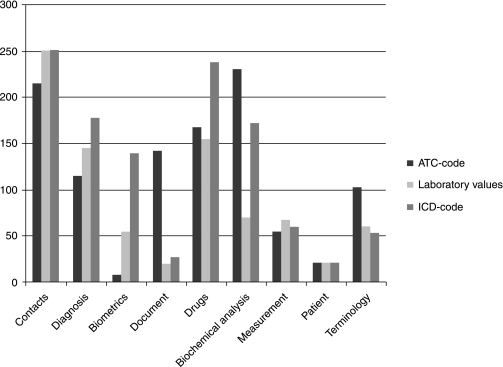
Data items (*n* = 3,045) compared to the original EMR distributed over nine CXP modules and by inclusion (ATC code, laboratory values, or ICD code).

In this second part of the evaluation 3,045 data items were manually compared by coherence and occurrence to their original EMR source. The data items originated from 63 EMRs: 21 included by ICD code (1,060 data items), 21 by ATC code (1,216 data items), and 21 by laboratory values (769 data items). Figure 3 illustrates the distribution over the nine CXP tables of controlled data items. Thirteen of the 3,045 data items could only be found in the extracted data and not in the original EMR. These 13 data items originated from the *Contacts* table. By using an administrator login, access was given to data that had been deleted in the original EMR. We then located all 13 data items, increasing the congruity from 99.6% to 100%.

## Discussion

The emergence of EMRs has made primary care patient data more accessible to pharmaceutical companies, policy-makers, and researchers through data mining. Registers of large populations have been generated to monitor expected and adverse drug effects, diseases, or health care expenditure. Data mining may be either semi-automated or automated. The semi-automated techniques find EMRs that meet the wanted criteria but cannot extract specified clinical information. Manual imputation by project employees or by caregivers at the PHCs is needed for this. The imputation is time-consuming, and there is a risk that it lags behind by local disturbances at the PHC. Automated extraction tools do not need manual imputation. This makes the process faster, cheaper, and independent of everyday clinical practice. If there is an inconsistency among caregivers or between PHCs in EMR documentation, the automated extraction tool may not find relevant data. This is explicatory of why registers generated by automated data extraction typically have not only a high coverage but also a high number of missing values (9–17).

The use of data extracted from EMRs for clinical surveillance and research is widespread. We found only one published evaluation of an automated extraction tool. Liljeqvist et al. (18) developed an extraction tool to find cases of influenza-like illness (ILI) by searching both in free text and in coded data. The sensitivity and specificity of extracting relevant ILI cases by search in free text were tested by using two public health physicians to study the EMRs of two PHCs during one week. The congruity of extracted material with its original source was not addressed. We have studied both the ability of CXP to extract relevant cases and manually compared extracted data to original data to address the congruity of the free text extraction. A direct comparison between different extraction tools is difficult to carry out since a new extraction tool needs to be developed for every EMR provider. A consensus between the EMR providers would open up possibilities for nation-wide (or even international) registers to be generated. Such registers would facilitate surveillance of clinical practice and utilization of health resources and stimulate epidemiological research of real-life clinical data.

### Concluding remarks

In summary, with a positive prediction value of 97.3%, a negative prediction value of 100%, and a congruity of 100% with the original source, automated data extraction proves to be a safe and feasible way to generate high-quality patient registers from primary care EMRs. Registers provided by automated EMR extraction are cost-effective tools to monitor treatment patterns, complications, and implementation of guidelines, as well as being a source for epidemiological and health economic research in a real-life setting.
